# Demographic Determinants of Disaster Preparedness Behaviors Amongst Tehran Inhabitants, Iran

**DOI:** 10.1371/currents.dis.976b0ab9c9d9941cbbae3775a6c5fbe6

**Published:** 2015-12-11

**Authors:** Mehdi Najafi, Ali Ardalan, Ali Akbarisari, Ahmad Ali Noorbala, Hossain Jabbari

**Affiliations:** Department of Disaster & Emergency Health, School of Public Health, Tehran University of Medical Sciences, Tehran, Iran; Department of Disaster & Emergency Health, National Institute of Health Research, Tehran University of Medical Sciences, Tehran, Iran; Department of Disaster Public Health, School of Public Health, Tehran University of Medical Sciences, Tehran, Iran; Harvard Humanitarian Initiative, Harvard University, Cambridge, MA, USA; Department of Health Management and Economics, School of Public Health, Tehran University of Medical Sciences, Tehran, Iran; Department of Psychiatry, Tehran University of Medical Sciences, Tehran, Iran; Department of Disaster Public Health, School of Public Health, Tehran University of Medical Sciences, Tehran, Iran; Department of Infectious Diseases and Tropical Medicine, School of Medicine, Tehran University of Medical Sciences, Tehran, Iran

## Abstract

Background: Tehran is vulnerable to natural hazards, especially earthquakes. Disaster preparedness behaviors (DPB) are measures that can mitigate the adverse consequences of disasters. Demographic factors affect DPB, however, the role of some of these factors is not still clear. By understanding these effects, disaster specialists could design interventions toward specific demographics. In the present study, we aimed to investigate demographic determinants of DPB.

Methods: This cross-sectional survey was conducted in August 2014. The target population included inhabitants of Tehran who were 18 years or older. A total of 1250 participants were selected randomly and interviewed using a standardized questionnaire.

Results: Results of our study showed that monthly income level, previous disaster experience, residential district and occupation are demographic factors that influence DPB significantly. However, disaster preparedness was not affected by gender, educational level, number of household members, home type, home ownership and being the head of household.

Conclusion: To promote DPB in Tehran, disaster specialists should focus on improving DPB in low-income and unemployed people, and individuals who live in high risk districts, especially in those who have not experienced disasters.

Key words: Disaster, Preparedness behavior, Demographic determinants.

## Introduction

Tehran, the capital of Iran, has been the place of some historically destructive earthquakes. Recent seismotectonic studies show that this city is located in a high seismic activity zone^1^^,^ 2 and it is probable that a large damaging earthquake will occur in Tehran in the near future^3^. Each of the 22 districts in Tehran has a different seismic hazard risk^4^. In cities like Tehran, natural hazards such as earthquakes cannot be prevented, but their damages can be mitigated by disaster preparedness behaviors (DPB) which denotes activities that people can do to protect their health against disasters. Factors influencing DPB include: risk perception^5^^-^ 8, preparedness perception^9^^-^ 11, critical awareness^8^^,^ 12 ^,^ 13, optimistic and normalization biases^14^^,^ 15, self-efficacy^9^^,^ 16 ^-^ 18, collective efficacy^19^, fatalism^7^^,^ 17 ^,^ 20 ^-^ 22, locus of control^7^^,^ 18 ^,^ 22, anxiety^8^^,^ 22 ^,^ 23, societal norms^24^, sense of community^25^, community participation and empowerment^26^^,^ 27, social trust^28^, perceived responsibility^6^^-^ 10, responsibility towards others^13^, coping style^9^^,^ 16 ^,^ 29 ^,^ 30, available resources^26^^,^ 31, and demographics.

One of the most important factors affecting DPB is demographics. Although there are studies indicating the role of demographics on disaster preparedness, however, more studies are required to shed more light on all aspects of this issue. On the other hand, the role of some demographic factors in DPB is still unclear. Therefore, it is important to identify the effect(s) of demographic factors like gender, age, occupation, income, education, previous disaster experience, home ownership, home type and residential district on DPB.

There are couple of studies which confirm the relationship between gender and DPB and many others with contradictory results^32^. For example, some studies strongly suggest that men engage in DPB more than women while some studies reject this hypothesis^33^^,^ 34. Similarly, some studies indicate that DPB increases with age^35^^,^ 36 while others showed that older people are less likely to engage in preparation^37^. Socioeconomic status has also been considered to be an indicator of preparedness levels in several studies. Russell, Goltz and Bourque found that survival preparedness, preparedness planning and hazard mitigation were associated with having a higher income while preparedness planning was associated with having a higher education^38^. Eisenman et al. found that having some college or trade school education was associated with increased odds of having emergency supplies^39^. However, Nguyen et al. found no association between socioeconomic variables and earthquake preparedness^40^. Comparably, certain studies have shown that disaster experiences affect DPB^6^^,^ 7 ^,^ 38 ^,^ 41 ^-^ 43. Several studies have also shown that previous experience is one of the determinants of preparedness^9^. However, a few studies have not confirmed such findings^44^. There are, however, just a few studies about the effect of other demographics like occupation, home ownership, home type and residential district on DPB.

To date, little research has focused on the demographic determinants of DPB in Tehran. In the present study, we evaluate the effects of some demographic factors on DPB amongst Tehran inhabitants.

## Materials and Methods

***Study population and sampling***

This cross-sectional survey was conducted in August 2014. The target population included inhabitants of Tehran who were 18 years and older. 1250 inhabitants were enrolled in the study through a random multistage sampling method from 22 districts in Tehran. The sample size for each district was calculated to be proportional to the size of the district populations. Firstly, after numbering the blocks, one of the blocks was selected randomly in each district. Secondly, moving in a clockwise direction from that corner, all houses up to the next corner were numbered and one of these, the first unit in the sample was also randomly chosen. Trained interviewers started from the first selected unit, filled the questionnaire based on the responses from individuals older than 18 years and capable of answering the questions. Then the next three units were systematically skipped and a person in the fifth household was interviewed and this continued until the end of the block. If the selected block did not include sufficient samples, the next block (in numerical order) was selected for completing the cluster.

The study was approved by the Tehran University of Medical Sciences Research Ethics Committee. Written consent was received from participants. We did not collect any identifying data.

***Questionnaire***

We used a standardized questionnaire to collect data. The questionnaire contained questions on demographic characteristics including gender, age, level of education, occupation, income, home ownership, home type, number of household members, previous disaster experience, being the head of a household and residential district. Another set of questions related to DPB based on the behavioral elements of public readiness index (PRI) was also included^45^. The validity and reliability of PRI have been shown in previous studies^45^. The PRI could be used in every emergency. The PRI measures how prepared persons and households are, and provides a practical “score” that assesses their readiness. The PRI is scored on a scale of 0 to 10 based on responses to 10 questions that examine key emergency preparedness knowledge (3 questions) and behavioral elements (7 questions)^45^. Since we aimed to study the DPB of participants, we used only the behavioral elements of the PRI as it examines only emergency preparedness behavior and it is scored on a scale of 0 to 7 (Table 1). The behavioral elements of the PRI are referred to as DPB index.

**Table 1. DPB index: The behavioral elements of the public readiness index (PRI):** 

Question
1) Have you actually prepared a disaster supply kit with emergency supplies like water, food and medicine that is kept in a designated place in your home?
2) Have you actually prepared a small kit with emergency supplies that you keep at home, in your car or where you work to take with you if you had to leave quickly?
3) Have you actually made a specific plan for how you and your family would communicate in an emergency situation if you were separated?
4) Have you actually established a specific meeting place to reunite in the event you and your family cannot return home or are evacuated?
5) Have you actually practiced or drilled on what to do in an emergency at home?
6) Have you actually volunteered to help prepare for or respond to a major emergency?
7) Have you actually taken first aid training such as CPR in the past five years?

***Data collection and analyses***

1250 participants were interviewed by trained interviewers in 22 city districts. The interviewers filled out the questionnaires during the interviews. 17 of the 1250 questionnaires were invalid because of missing data and so were excluded from subsequent analyses. Data were grouped according to sex, age, education, occupation, home ownership, home type, number of household members, previous disaster experience, being head of a household, income and residential district. The grouped data were subsequently statistically analyzed using independent Student's t-test, one way ANOVA, and the stepwise multiple regression analysis to compare means of the DPB score among different groups and to identify the predominant factors influencing DPB.

## Results

Table 2 shows the demographic characteristics of the study participants. 37.7% of participants were female while 62.3% of them were male. The mean age of all participants was 44.14 (SD = 12.53). 71.5% of participants had high school or higher education. 34.5% of participants were currently unemployed (including housewives, students, retired and jobless participants). 54% of participants were homeowners with most of them living in apartments (82.5%). Only 16.5% of the households had more than 4 members. 41.6% of the respondents had experienced at least one disaster in the past 20 years. 83.7% of participants were heads of households. 68.1% of responders lived in the high or medium risk districts of Tehran. Most of the participants (65%) reported that were low income earners.

**Table 2. Participant characteristics:** 

Variable	Groups	Frequency	Percent
Sex	Male	768	62.3
	Female	465	37.7
Age	18-34	384	28.2
	35-44	357	29
	45-54	261	21.2
	>55	267	21.7
Education level	Illiterate	81	6.6
	Less than high school	270	21.9
	High school	426	34.5
	More than high school	456	37
Occupation	Currently unemployed (housewife, retired, student or jobless)	426	34.5
	Currently employed	807	65.5
Home ownership	Owner	666	54
	Tenure	567	46
Home type	Apartment	1017	82.5
	House	216	17.5
Number of household members	≤4	1029	83.5
	>4	204	16.5
Previous disaster experience	Yes	513	41.6
	No	720	58.4
Being head of household	Yes	1032	83.7
	No	201	16.3
Residential district	High risk	381	30.9
	Medium risk	459	37.2
	Relatively low risk	393	31.9
Monthly income level	Low (less than 20 million Iranian Rials)	801	65
	Middle (20-40 million Iranian Rials)	354	28.7
	High (more than 40 million Iranian Rials)	78	6.3

Table 3 shows the DPB scores for the study participants. 90% of respondents had DPB score less than or equal to 4. It also shows that 43.1% of the respondents had not done anything to protect themselves from possible future disaster. The mean DPB score was 1.55 (SD = 1.93).

**Table 3. DPB scores for the study participants:** 

DPB score	Frequency	Percent	Cumulative percent
0	531	43.1	43.1
1	246	20	63
2	147	11.9	74.9
3	99	8	83
4	87	7.1	90
5	52	4.1	94.2
6	27	2.2	96.4
7	45	3.6	100

Independent sample t-test was used to define any significant difference between the two independent groups (Table 4). This analysis showed that men were more engaged in DPB than women. It also showed that the participants who experienced disasters are more prepared compared to those who had no such experience. The people who were heads of households had significantly more DPB than those who were not. Currently, employed participants had significantly more DPB scores than unemployed ones. The DPB score did not correlate with home ownership, home type and the number of household members.

**Table 4. Independent sample t-test for differences between the groups:** 

Variable	Groups	N	Mean of DPB score	SD	t	Sig. (2-tailed)	95% CI
Gender					4.680	0.000	0.295-0.720
	Male	768	1.75	2.02			
	Female	465	1.24	1.73			
Head of the household					2.383	0.018	0.056-0.592
	Yes	1032	1.61	1.96			
	No	201	1.28	1.72			
Occupation					4.918	0.000	0.322-0.771
	Currently employed	807	1.74	1.98			
	Currently unemployed (housewife, retired, student or jobless)	426	1.20	1.72			
Home ownership					0.342	0.733	-0.179-0.254
	Owner	666	1.57	1.87			
	Tenure	567	1.53	1.99			
Home type					0.123	0.902	-0.266-0.302
	Apartment	216	1.57	1.87			
	House	1017	1.55	1.99			
Number of household members					0.086	0.931	-0.278-0.303
	≤4	1029	1.56	1.91			
	>4	204	1.54	2.02			
Previous disaster experience					10.517	0.000	0.962-1.404
	Yes	513	2.25	2.19			
	No	720	1.06	1.54			

One-way analysis of variance (ANOVA) was used to test for the statistical significance of group differences (Table 5). Statistically significant group differences were observed for the participants’ DPB in different age groups. Post-hoc Tukey's tests showed that the DPB score was significantly greater for those who were 35-44 years old compared to those who were more than 55 years of age. There were significant group differences for DPB score in different districts. A post-hoc Tukey's test revealed that the DPB score in low risk districts was significantly higher than in high and medium risk districts. There was a positive correlation between the DPB score and income. Post-hoc Tukey's test revealed a significantly higher increase in the DPB score in high and mid-income participants compared with that in low-income participants. Statistically significant DPB score group differences were not observed in participants of the different educational groups.

**Table 5. One-way ANOVA for differences between the groups:** 

Variable	Groups	N	Mean of DPB score	SD	df	F	Sig.
Age					3	3.812	0.000
	18-34	384	1.47	1.73			
	35-44	357	1.84	2.17			
	45-54	261	1.45	1.90			
	>55	267	1.38	1.84			
Education level					3	0.537	0.657
	Illiterate	81	1.33	2.17			
	Less than high school	270	1.51	1.84			
	High school	426	1.56	1.87			
	More than high school	456	1.61	1.99			
Residential districts					2	14.847	0.000
	High risk	381	1.31	1.76			
	Medium risk	459	1.39	1.98			
	Relatively low risk	393	1.98	1.95			
Monthly income level					2	74.695	0.000
	Low	801	1.10	1.42			
	Middle	354	2.31	2.34			
	High	78	2.85	2.66			

Stepwise multiple regression analyses showed monthly income level, previous disaster experience, residential district and occupation as four significant demographic determinants for DPB. They explained 16.2% of the variance in DPB score. Monthly income level and previous disaster experience explained 10.4 and 4.6% of the variance in DPB score, respectively. Residential district accounted for 0.8% while occupation explained 0.4% of the variance (Table 6). This means that only 16.2 per cent of the variation in DPB in the sample is due to these four independent variables. The poor correlation between these variables and DPB indicates that these variables are not very strong to predict DPB.

**Table 6. Stepwise multiple regression analysis showing the relative contribution of each variable to predict DPB score:** ** p<0.001; * p<0.05

Predictors	R squre	R squre change	F change
Monthly income level	0.104	0.104	143.625^∗∗^
Monthly income level, Previous disaster experience	0.150	0.046	66.604^∗∗^
Monthly income level, Previous disaster experience, Residential district	0.158	0.008	11.257^∗^
Monthly income level, Previous disaster experience, Residential district, Occupation	0.162	0.004	5.711^∗^

## Discussion

The main purpose of this research was to study some demographic determinants of disaster preparedness behaviors (DPB) among Tehran inhabitants. Our findings showed that DPB was mainly affected by monthly income level, previous disaster experience, residential district and occupation.

Like our study, previous researches found that people with high income are more prepared than low income earners^46^^,^ 47. It could be explained by the fact that people with higher income are expected to have more access to qualified properties and live in more disaster resistant areas, while it is reverse for people with lower income^47^. It is also pointed out that poorer people are less likely to mitigate the effects of hazards because they lack a sense of personal control over potential outcomes^48^.

”Previous disaster experience” was another determinant of DPB considered in this study. The results are again consistent with some previous studies^42^^,^ 43. Past disaster experience may influence individuals to collect more information about disasters and as a result implement them for better disaster preparedness. In addition, a person who experiences a disaster probably has more risk perception than one who has not been exposed to such an experience. Risk perception can be defined as how much risk individuals perceive to be caused by a hazard or disaster^49^.

Like Taghizadeh et al.’s results^50^, our study showed that people in districts with low earthquake risk are more prepared than people living in districts with high earthquake risk. This again could be explained by the lower socioeconomic level of people living in the high risk districts. In addition, people with higher socioeconomic level are expected to be more educated and well-informed and so will have more risk perception. However, as we have not examined the participants' risk perception in this study, we could not confirm this hypothesis clearly.

In the present study, we also found that employed people are more prepared than unemployed individuals. Occupational characteristics may impact individuals’ real or perceived risk, their access or receptivity to information and their ability to carry out preparedness measures^51^.

Although significant association was found between income level, past disaster experience, residential area and occupation with DPB, the poor correlation between these variables and DPB shows that DPB also depends on factors other than these variables. As it was pointed out above, these variables accounted for only 16.2% of the variance in DPB which indicates that other factors need to be considered to further analyze the total DPB score of Tehran inhabitants. Some of these factors were referred to in the introduction.

Similar to our study and in other studies in other countries, gender was not a significant factor for DPB^52^. In Tehran, this issue may be due to an equality between men and women in making the same decision in disaster preparedness^33^. However, some of the studies showed that men have a higher DPB than women^33^^,^ 34. In these cases, women may be more unprepared than men due to socially determined differences in roles and responsibilities between them. This could also be because of inequalities between them in terms of decision-making power, participation in emergency preparedness organizations and access to resources^53^^,^ 54. Nevertheless, some studies have shown that women perceive disaster events or threats as more serious and risky than men do, and they are generally involved in more mitigation and preparedness activities than men, particularly in activities centered inside the house^54^.

Our study showed that age is not a significant determinant of DPB. Mileti and Darlington and Nguyen et al. like ours, found that age is unrelated to taking preparedness actions^40^^,^ 55. However, Lindell and Perry and also Eisenman et al. showed that age is related to how people respond to risk-related messages^39^^,^ 56. Some researchers also stated that older people are less prepared due to an optimistic bias; because they have survived previous disasters without injury or damage^57^. On the contrary, some studies illustrated that older individuals were significantly better prepared than younger ones^58^. It was also found that older adults were emotionally resilient^59^.

Our study showed that education was not a determinant of DPB in inhabitants of Tehran. Unlike our findings, some studies showed that formal education is a determinant of DPB^60^. It is believed that formal education can promote disaster preparedness because education enhances individual cognitive and learning skills, as well as access to information^61^.

Home ownership was not also a DPB determinant. Like our study, others did not find a significant relationship between home ownership and preparedness^62^. Unlike ours, a number of studies have shown that homeowners are more likely to be prepared than those in rented apartments^46^. This could be due to the responsibilities that owners take and renters prefer to avoid^63^.

Having more household members, especially children at home could contribute to how household heads prepare for a disaster^64^. Nevertheless, our study shows that neither number of household members, nor being a household head was a determinant of DPB.

## Conclusion

Our findings show that monthly income level, previous disaster experience, residential district and occupation influence disaster preparedness behaviors of Tehran inhabitants. Therefore, in order to achieve higher levels of individual disaster preparedness, it is crucial that governmental officials, emergency agencies, community leaders and educators pay more attention to low-income individuals, unemployed people, and individuals who live in high risk districts, especially those have not experienced disasters.

## Competing Interests

The authors have declared that no competing interests exists.
